# Long-term follow-up of recovered MPN patients with COVID-19

**DOI:** 10.1038/s41408-021-00509-0

**Published:** 2021-06-16

**Authors:** Tiziano Barbui, Alessandra Iurlo, Arianna Masciulli, Alessandra Carobbio, Arianna Ghirardi, Giuseppe Rossi, Claire Harrison, Alberto Alvarez-Larran, Elena Maria Elli, Jean-Jaques Kiladjian, Mercedes Gasior Kabat, Alberto Marin Sanchez, Francesca Palandri, Marcio Miguel Andrade-Campos, Alessandro Maria Vannucchi, Gonzalo Carreno-Tarragona, Petros Papadopoulos, Keina Quiroz Cervantes, Maria Angeles Foncillas, Maria Laura Fox, Miguel Sagues Serrano, Elisa Rumi, Santiago Osorio, Giulia Benevolo, Andrea Patriarca, Begona Navas Elorza, Valentin Garcia-Gutierrez, Elena Magro Mazo, Francesca Lunghi, Massimiliano Bonifacio, Valerio De Stefano, Juan Carlos Hernandez-Boluda, Emma Lopez Abadia, Anna Angona, Blanca Xicoy Cirici, Marco Ruggeri, Steffen Koschmieder, Marta Anna Sobas, Beatriz Cuevas, Daniele Cattaneo, Rosa Daffini, Marta Bellini, Natalia Curto-Garcia, Marta Garrote, Fabrizio Cavalca, Lina Benajiba, Beatriz Bellosillo, Paola Guglielmelli, Oscar Borsani, Silvia Betti, Silvia Salmoiraghi, Alessandro Rambaldi

**Affiliations:** 1 FROM Research Foundation, Papa Giovanni XXIII Hospital, Bergamo, Italy; 2 Hematology Division, Foundation IRCCS Ca’ Granda Ospedale Maggiore Policlinico, Milan, Italy; 3 Spedali Civili, Brescia, Italy; 4 Guy’s and St. Thomas’ NHS Foundation Trust, London, UK; 5 Hospital Clinic de Barcelona, Barcelona, Spain; 6 Hematology Division and Bone Marrow Transplant Unit. San Gerardo Hospital, ASST Monza, Monza, Italy; 7 Hospital Saint-Louis, Paris, France; 8 Hospital Universitario la Paz, Madrid, Spain; 9 Hospital General Universitario de Albacete, Albacete, Spain; 10 IRCCS Azienda Ospedaliero-Universitaria di Bologna, Bologna, Italy; 11 Hospital del Mar, Barcelona, Spain; 12 Center Research and Innovation of Myeloproliferative Neoplasms (CRIMM), Department of Experimental and Clinical Medicine, Azienda Ospedaliera Universitaria Careggi, University of Florence, Florence, Italy; 13 Hospital Universitario 12 de Octubre, Madrid, Spain; 14 Hospital Clinico San Carlos, Madrid, Spain; 15 Hospital Universitario de Mostoles, Madrid, Spain; 16 Hospital Universitario Infanta Leonor, Madrid, Spain; 17 Department of Hematology, Vall d’Hebron Institute of Oncology (VHIO), Vall d’Hebron Hospital Universitari, Vall d’Hebron Barcelona Hospital Campus, C/ Natzaret, 115-117, 08035 Barcelona, Spain; 18 ICO L’Hospitalet-Hospital Moises Broggi, Sant Joan Despì, Barcelona, Spain; 19 Department of Molecular Medicine, University of Pavia, Pavia, Italy; 20 H30,ospital Gregorio Maranon, Madrid, Spain; 21 AOU Città della Salute e della Scienza di Torino, Torino, Italy; 22 AOU Maggiore della Carità, Novara, Italy; 23 Hospital Moncloa, Madrid, Spain; 24 Hospital Ramon y Cajal, IRYCIS, Madrid, Spain; 25 Hospital Universitario Principe de Asturias, Alcalà de Henares, Madrid, Spain; 26 IRCCS Ospedale San Raffaele, Milano, Italy; 27 Ospedale Policlinico “G.B. Rossi”, Borgo Roma, Verona, Italy; 28 Fondazione Policlinico “A. Gemelli” IRCCS, Rome, Italy; 29 Hospital Clinico Universitario, INCLIVA, Valencia, Spain; 30 Hospital General de Elche, Elche (Alicante), Alicante, Spain; 31 ICO Girona Hospital Josep Trueta, Girona, Spain; 32 Hospital Germans Trias i Pujol, Badalona (Barcelona), Barcelona, Spain; 33 Ospedale San Bortolo, Vicenza, Italy; 34 Department of Hematology, Oncology, Hemostaseology, and Stem Cell Transplantation, Faculty of Medicine, RWTH Aachen University, Aachen, Germany; 35 Department of Hematology, Blood Neoplasms and Bone Marrow Transplantation, Wroclaw Medical University, Wrocław, Poland; 36 Hospital Universitario de Burgos, Burgos, Spain; 37 ASST Papa Giovanni XXIII, Bergamo, Italy; 38 Università degli Studi di Milano, Milano, Italy

**Keywords:** Myeloproliferative disease, Infectious diseases

**Dear Editor,**

During the first wave of SARS-CoV-2 infection a European observational study was launched under the auspices of the European Leukemia Net (ELN), aiming at gathering information about the clinical epidemiology of COVID-19 in patients with chronic myeloproliferative neoplasms (MPN-COVID study).

Thirty-eight hematologic centers from Italy, Spain, Germany, France, United Kingdom and Poland, participated in the study and enrolled 180 consecutive patients with WHO-diagnosis of essential thrombocythemia (ET; *n* = 60), polycythemia vera (PV; *n* = 58), prefibrotic-myelofibrosis (pre-PMF; *p* = 23) and overt primary myelofibrosis (PMF; *n* = 39) who developed COVID-19 from February 15 to May 31, 2020. During the acute phase of the infection, in-hospital mortality affected almost 30% of 175 evaluable patients and the most vulnerable MPN subgroup was overt PMF (mortality 48%) [1]. Another result deriving from this cohort concerned the thrombosis incidence, found significantly elevated in ET, where it reached almost 20% vs. 5% in PV and PMF, respectively [2].

At present, there is no information on the clinical condition of MPN patients discharged after COVID-19. Although SARS-CoV-2 infection has a variable clinical severity and mainly manifests itself as a respiratory syndrome, accumulating data revealed damage of hematopoietic system and vascular endothelium. This damage can persist even after the acute phase of infection [3]. Post COVID-19 related consequences could be more frequent in clonal diseases such as MPN, whose natural history is marked by vascular complications and inherent risk of clonal evolution into myelofibrosis, myelodysplasia (MDS) and acute myeloid leukemia (AML).

In this paper, we report the events that occurred in 125 of the 175 patients (71%), enrolled in MPN-COVID study, who survived to the acute phase of the infection. Participating centers were required to report in pre-established e-CRFs patient characteristics and outcomes collected after at least 6 months after COVID-19 infection recovery. The study was approved by the single center Ethical Committees.

## Patient characteristics

### Before and at COVID-19

In Table S1, surviving patients examined in the last-follow-up before COVID-19 (median: 1.4 months; interquartile range [IQR]: 0.8–3.0) presented blood counts and clinical picture consistent with the chronic phase of their respective MPN subtypes.

The great majority experienced an infection of moderate severity (94.4%) and patients were managed at home (*n* = 38; 30.4%) or in regular wards (*n* = 80; 64%), whereas only seven (5.6%) were in need of intensive care unit (ICU) admission. COVID-19 directed therapy included antiviral agents (*n* = 43; 35.8%) and in 28 patients (23.7%) corticosteroids. High C-reactive protein (CRP), neutrophils on lymphocytes (N/L) ratio levels and D-Dimer increase marked the clinical course. We point out that antithrombotic drugs including low molecular weight heparin (LMWH) and oral anticoagulants were prescribed in 54% and 2.5%, respectively, of these 125 patients and that, in spite of this treatment, venous thromboembolism was diagnosed in 7 patients (5.6%) and a stroke in a single case (0.8%).

### Post-COVID

The blood count values after 6 months from the resolution of the infection are presented in Table S1 as medians and interquartile ranges and do not show substantial alterations. Of note, chest CT-scan was abnormal in 69% of 19 examined patients and in 10% patients the O_2_ saturation was less than 95%. Among the laboratory tests, we noted the persistence of increased inflammation markers (i.e., CRP ≥ 0.8 mg/dl in 56% of cases; N/L ratio ≥ 3 in 48%; D-Dimer ≥500 ng/mL in 18%), as reported in the general population after COVID-19 [4]. Cytoreductive therapy was used in 80% of cases and antithrombotic therapy included antiplatelet agents in 67.5% of cases and anticoagulants in 17.5%.

## Outcomes post-COVID-19

### Symptoms

A long-term follow-up study of these patients at 6 months post infection revealed ongoing symptoms in a third of the patients (Fig. S1). Among these, fever, cough, and dyspnea were the most commonly reported during the acute infection but largely remitted in the following 6 months. It has been reported that the persistence of symptoms is more frequent in patients over the age of 60 and in our cases, with a median of 70 years of age, it was also associated with persistence of inflammatory markers in more than half of them. Overall, these findings suggest a slow recovery after the acute phase of infection as also observed in other series of non-MPN patients [5,6,].

### Major thromboses and bleedings

**Fig. 1:**
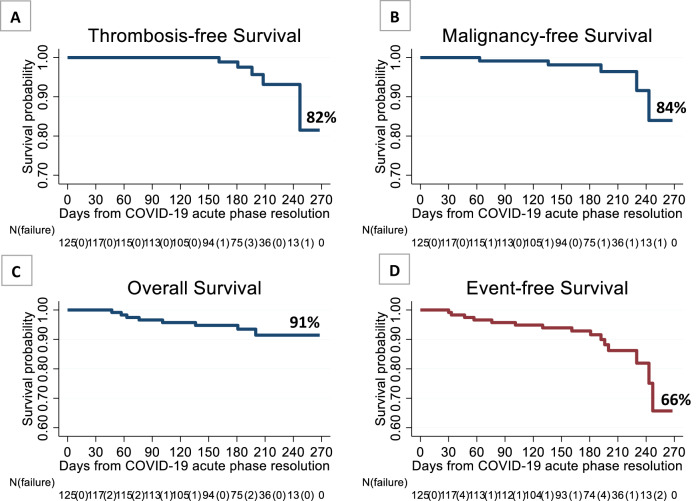
Major outcomes at 6-months follow-up after COVID-19 recovery. Kaplan–Meier survival estimates from COVID-19 recovery of (**A**) Thrombosis-free, (**B**) Malignancy-free, (**C**) Overall survival and (**D**) Event-free survival.

Major thrombosis was registered in five patients (4%); in one of them, massive fatal intestinal ischemia occurred (Table 1). None of these patients experienced thrombosis during COVID-19. The acute infectious disease of these patients was managed in the ordinary wards and, after discharge, antithrombotic therapy with LMWH was only used in a single patient who developed peripheral arterial thrombosis. Of the four patients receiving cytoreductive drugs, three were receiving ruxolitinib for PMF or ET, and the fourth was on hydroxyurea (HU). It is interesting to know that these events occurred after 5 months after the infection subsided, as it is well illustrated by the Kaplan Meyer thrombosis-free survival curve of Fig. 1A.

**Table 1:** Main characteristics of patients with major outcomes at the 6-month follow-up after COVID-19 recovery.

THROMBOSIS (*N* = 5)	Fatal event	MPN	Age	COVID-19 acute phase disposition	Cytoreduction	Anticoagulation
Intestinal ischemia	Yes	MF	71.7	Regular ward	No	No
Splenic infarction	No	MF	72.3	Regular ward	Ruxolitinib	No
DVT (legs) + PE	No	ET	61.5	Regular ward	Ruxolitinib	No
Acute myocardial infarction	No	PV	80.6	Regular ward	Hydroxyurea	No
Pheripheral arterial thrombosis	No	MF	75.4	Regular ward	Ruxolitinib	Yes

MALIGNANCY (*N* = 5)	Fatal event	MPN	Age	COVID-19 acute phase disposition	Cytoreduction	MPN disease duration
AML	Yes	MF	49.3	ICU	Hydroxyurea	5.9 years
AML	No	ET	78.3	Regular ward	Hydroxyurea	6.0 years
AML	No	Pre-PMF	82.1	Regular ward	Hydroxyurea	3.8 years
Non-Hodgkin lymphoma	No	ET	60.0	Regular ward	Hydroxyurea	8.6 years
Progression of Parotid Carcinoma	Yes	MF	77.3	Regular ward	Ruxolitinib	21.7 years

DEATH CAUSES (*N* = 8)	Fatal event	MPN	Age	COVID-19 acute phase disposition	Cytoreduction	MPN disease duration
AML	–	MF	49.3	ICU	Hydroxyurea	5.9 years
Progression of solid cancer (parotid)	–	MF	77.3	Regular ward	Ruxolitinib	21.7 years
Suspected lung cancer	–	MF	80.9	Regular ward	unk	10.9 years
Multi Organ Failure	–	ET	85.7	Regular ward	Hydroxyurea	5.8 years
Thrombosis	–	MF	71.7	Regular ward	No	10.2 years
Heart failure	–	MF	82.4	Home	Unknown	24.7 years
Heart failure	–	Pre-PMF	88.7	Home	No	8.8 years
Unknown	–	ET	87.0	Regular ward	Unknown	0.8 years

A single patient with PMF experienced a recurrence of gastrointestinal bleeding requiring blood transfusions ~3 months after discharge.

These findings are difficult to compare with the data reported in the general population in the post-infection period. A number of studies reported varying incidences of 1–5% of venous thromboembolism in patients after discharge, depending on the underlying disease, comorbidity, and concomitant antithrombotic prophylaxis [7–9]. Clearly, one factor that may explain some of these differences is the observation time after infection. In our cases with MPN, no event was recorded in the first 5 months but, instead, they occurred later, during the last period of our observation. Notably, our patients were not on LMWH prophylaxis during this time, which could possibly have reduced these vascular complications [10].

### Malignancies

#### Acute myeloid leukemia

AML was diagnosed in 3 patients by morphology, immunophenotyping, cytogenetics and genetics including next generation sequencing (NGS) (Table S2).

Patient #1, with PMF *CALR*-mutated, upon progression showed numerous recurrent karyotype abnormalities, and the presence of multiple genetic lesions typically associated with progression in AML was documented.

Patient #2, with ET *JAK2*V617F (variant allele frequency [VAF] 31%), upon progression to AML showed a karyotype characterized by the presence of an additional marker, and genetic lesions associated with AML evolution were revealed by NGS.

Patient #3, during chronic phase of pre-PMF, in addition to the *MPL* mutation (VAF 1%) also showed the presence of high-risk genetic lesions [11]. On progression, a complex karyotype was found and the molecular profile documented additional genetic lesions of *TP53* (VAF 91%) and *RUNX1* (VAF 44%).

While it is conceivable that the COVID-19 hyperinflammatory state associated with the acute phase of the infection and persisting even after recovery could have accelerated disease progression in patient #3, the molecular profile of the other two patients did not suggest such rapid progression to AML.

#### Large B-cell Non-Hodgkin lymphoma

Large B-cell non-Hodgkin lymphoma was diagnosed in a single case; the tumor developed predominantly in the brain and patient is currently alive on chemotherapy.

#### Parotid cancer

The patient had a rapid evolution post-COVID-19, whereas it was stable before. The tumor showed an unexpected aggressiveness leading to death of the patient.

These five malignant events were diagnosed as early as the second post-COVID-19 month and the probability of their occurrence was projected to 20% after 8 months. To our knowledge, the onset of neoplastic events in the immediate post-COVID-19 period in patients with MPN has not been reported so far. Given the low number of events, we were unable to investigate any risk factors. We can speculate that both MPN and COVID-19 share overlapping inflammatory mechanisms that may have favored the disease progression of malignant subclones already present in the chronic phase of MPN.

### Mortality

Deaths occurred in eight patients after 9 months and the causes are listed in Table 1. Kaplan–Meier curve indicated that probability of death was 9% (Fig. 1C).

Event-free survival after 9 months, including freedom from thrombosis, malignancies and death, was 66% in the 125 surviving patients, who were followed-up for a median of 6 months post-COVID-19 recovery (Fig. 1D).

This multicenter European study, although with a relatively small number of patients, provides a descriptive analysis of MPN patients who survived after COVID-19. We here report a diversity of complications that further increase mortality and morbidity of MPN patients in the post-COVID-19 period. Indeed, 40% of these patients, when followed-up over 6 months after the acute COVID-19 illness subsided, experienced both fatal and non-fatal events.

Our research indicates that the health consequences of COVID-19 extend far beyond acute infection and suggest larger, multi-center analyses to augment and expand our observations. These signals should induce careful surveillance in all patients with MPN regardless of the severity of acute SARS-CoV-2 infection.

## Supplementary information

Supplemetary material
