# Supplementary material for: Long-term follow-up of recovered MPN patients with COVID-19
Source: Blood Cancer J. 2021 Jun 16;11(6):115. doi: 10.1038/s41408-021-00509-0 (PMC8208062; doi:10.1038/s41408-021-00509-0)
Supplement: Supplementary file 1 — Supplemetary material [file 41408_2021_509_MOESM1_ESM.docx]

## **SUPPLEMENTARY MATERIAL**

### Table S1. Demographic, clinical and laboratory characteristics of patients before, at and after COVID-19 diagnosis

| **CHARACTERISTIC** | **N non missing** | **VALUE** |
| --- | --- | --- |
| ***Pre-COVID-19 diagnosis*** | | |
| Months before COVID-19 onset, median (IQR) | 125 | 1.4 (0.8-3.0) |
| *MPN diagnosis* | 125 |  |
| ET |  | 38 (30.4%) |
| PV |  | 37 (29.6%) |
| MF |  | 36 (28.8%) |
| pre-PMF |  | 14 (11.2%) |
| Age, median (IQR) | 125 | 68.9 (57.2-78.6) |
| *Sex* | 125 |  |
| Female |  | 58 (46.4%) |
| Male |  | 67 (53.6%) |
| Comorbidities (at least one) | 125 | 88 (70.4%) |
| Palpable splenomegaly | 107 | 28 (26.2%) |
| *Laboratory parameters, median (IQR)* |  |  |
| Hemoglobin, g/dL | 117 | 13.0 (11.4-14.2) |
| Hematocrit, % | 106 | 40.6 (35.0-43.2) |
| White blood cells count, x10^9^/L | 116 | 7.0 (5.4-9.1) |
| Platelets count, x10^9^/L | 116 | 336.5 (234.0-508.0) |
| *MPN directed treatments* | 125 |  |
| Hydroxyurea |  | 60 (48.0%) |
| Ruxolitinib |  | 25 (20.0%) |
| Anagrelide |  | 5 (4.0%) |
| Interferon |  | 4 (3.2%) |
| ***At COVID-19 diagnosis*** | | |
| Age, median (IQR) | 125 | 69.5 (57.6-78.9) |
| Years from MPN diagnosis, median (IQR) | 125 | 6.0 (3.1-10.4) |
| *Patient disposition* | 125 |  |
| Home |  | 38 (30.4%) |
| Regular ward |  | 80 (64.0%) |
| Intensive care unit |  | 7 (5.6%) |
| *Oxygen supplementation* | 125 |  |
| Non-invasive |  | 53 (42.4%) |
| Invasive |  | 6 (4.8%) |
| *Laboratory parameters, median (IQR)* |  |  |
| Hemoglobin, g/dL | 101 | 12.6 (10.4-13.5) |
| Hematocrit, % | 96 | 39.0 (32.0-42.8) |
| White blood cells count, x10^9^/L | 102 | 6.4 (4.5-9.2) |
| Platelets count, x10^9^/L | 100 | 260.5 (170.5-437.5) |
| Neutrophil/Lymphocyte ratio | 90 | 5.0 (3.3-8.6) |
| C-reactive protein, mg/dL | 77 | 66.0 (17.2-118.4) |
| D-Dimer, ng/mL | 61 | 597.0 (305.0-1392.0) |
| *COVID-19 directed treatment* | 125 |  |
| Steroids | 118 | 28 (23.7%) |
| Antibiotics | 117 | 77 (65.8%) |
| hydroxyc | 122 | 73 (59.8%) |
| Antivirals | 120 | 43 (35.8%) |
| Lopinavir/Ritonavir |  | 33 (27.5%) |
| Other |  | 7 (5.8%) |
| Experimentals | 123 | 11 (8.9%) |
| Tocilizumab |  | 7 (5.7%) |
| Ruxolitinib |  | 2 (1.6%) |
| Other |  | 2 (1.6%) |
| Antithrombotics | 121 | 70 (57.9%) |
| Low molecular weight heparin |  | 66 (54.5%) |
| DOACs |  | 2 (1.7%) |
| Warfarin |  | 1 (0.8%) |
| ***At 6-month follow-up after COVID-19 recovery*** | | |
| Days since hospital discharge/home resolution, median (IQR) | 125 | 185.0 (150.0-215.0) |
| Oxygen saturation, median (IQR) | 40 | 97.0 (95.5-98.0) |
| *Laboratory parameters, median (IQR)* |  |  |
| Hemoglobin, g/dL | 113 | 13.0 (11.3-14.2) |
| Hematocrit, % | 108 | 39.7 (35.0-43.7) |
| White blood cells count, x109/L | 113 | 7.3 (5.8-9.1) |
| Platelets count, x109/L | 111 | 320.0 (221.0-465.0) |
| Neutrophil/Lymphocyte ratio | 98 | 2.8 (2.0-4.7) |
| C-reactive protein, mg/dL | 41 | 1.2 (0.3-6.0) |
| D-Dimer, ng/mL | 22 | 361.5 (270.0-464.0) |
| *Instrumental examinations* |  |  |
| Chest X-ray investigation | 23 |  |
| Normal |  | 16 (69.6%) |
| Abnormal |  | 7 (30.4%) |
| Chest CT scan investigation | 19 |  |
| Normal |  | 6 (31.6%) |
| Abnormal |  | 13 (68.4%) |
| *Clinical evaluation* |  |  |
| Palpable splenomegaly | 98 | 30 (30.6%) |
| Cm below costal margin, median (IQR) | 30 | 3.0 (2.0-7.0) |
| Hepatomegaly | 101 | 9 (8.9%) |
| Lymphoadenopathy | 100 | 1 (1.0%) |
| Localization |  |  |
| Thorax |  | 1 (1.0%) |
| Need of blood transfusion | 110 | 13 (11.8%) |
| No. of tranfusions, median (IQR) | 13 | 6.0 (3.0-18.0) |
| *MPN directed treatments* |  |  |
| Hydroxyurea | 114 | 57 (50.0%) |
| Ruxolitinib | 114 | 27 (23.7%) |
| Interferon | 114 | 5 (4.4%) |
| Anagrelide | 114 | 3 (2.6%) |
| Antiplatelets | 114 | 77 (67.5%) |
| Aspirin |  | 68 (59.6%) |
| Clopidrogel/Tiklid |  | 9 (7.9%) |
| Anticoagulants | 114 | 20 (17.5%) |
| VKA |  | 8 (7.0%) |
| DOACs |  | 6 (5.3%) |

### Figure S1. Symptoms at and 6-months after acute COVID-19 phase

### Table S2. Laboratory and genetic characteristics of 3 MPN patients evolved into AML after COVID-19 recovery

| Patient characteristics | Patient #1 | Patient #2 | Patient #3 |
| --- | --- | --- | --- |
| *Primary MPN Diagnosis* | MF | ET | Pre-PMF |
| *Sex (Female/Male)* | M | F | M |
| *Blood counts at last chronic phase control* |  |  |  |
| *Hemoglobin (g/dl)* | 12.8 | 11.8 | 13.1 |
| *White Blood Cells (X10^9^/L)* | 6.07 | 5.5 | 15 |
| *Blasts (%)* | NA | NA | NA |
| *Platelets (X10^9^/L)* | 642 | 300 | 671 |
| *Blood counts at progression* |  |  |  |
| *Hemoglobin (g/dl)* | 7.5 | 8.2 | 9.3 |
| *White Blood Cells (X10^9^/L)* | 6.8 | 1.8 | 25.3 |
| *Blasts (%)* | NA | 1 | NA |
| *Platelets (X10^9^/L)* | 91 | 239 | 98 |
| *Bone Marrow Blasts (%)* | NA | 12 | NA |
| *Karyotype* |  |  |  |
| *During Chronic Phase* | NA | NA | NA |
| *At progression* | 45,XY,der(3;16)(q10;p10),-5,t(6;17)(p25;q11),+8,dic(8;9)(p21;p24),add(14)(q24),der(?)t(?;3)(?;q12)[25].ish der(3;16)(wcp3+),der(?)t(?;3)(?;q12)(wcp3+)[5],der(3;16)(RPN1+,MECOM+),der(?)t(?;3)(?;q12)(RPN1+,MECOM+)[5],der(14)t(5;14)(p13;q24)(wcp5+),der(?)t(?;3)ins(?;5)(wcp5+)[5],der(14)t(5;14)(p13;q24)(D5S23/D5S721+) [5] | 47,XX,+mar[11]/46,XX[17] | 46,XY,add(6)(p25)[6]/47,XY,-5,add(6)(p25),+r,+mar[4]/46,XY,del(1)(q32),5,add(8)(p23),-12,add(13)(p13),+mar1,+mar2,[8]/46,XY[2] |
| *Molecular Genetics* |  |  |  |
| *During Chronic Phase* | ***CALR*** c.1099_1150del, VAF 42% | ***JAK2*** V617F, VAF 31% | ***ASXL1*** c.1934dupG, VAF 15%, ***IDH1*** c.395G>A, VAF 1%, **IDH2** c.440T>A, VAF 15%, ***MPL***  c.1544G>T, VAF 1%, ***SRSF2*** c.284C>A, VAF 22% |
| *At progression* | WT for the investigated variants (*JAK2* V617F and exon 12, *NPM1*, *FLT3*, hot spot mutations *IDH1*/*2*) | ***DNMT3A*** p.Y528_Q534dup, VAF 45%***, IDH2*** p.R140Q, VAF 32%***, RUNX1*** p.F131V, VAF 3%,***SH2B3*** p.L414R, VAF 29%, ***STAG2*** c.1638+2T>C, VAF 4%, ***JAK2*** V617F<1% | ***ASXL1*** c.1934dupG, VAF 35%, ***IDH1*** c.395G>A, VAF 44%, ***MPL***  c.1544G>T, VAF 59%,  ***TP53*** c.524G>A, VAF 91%,  ***RUNX1*** c.320G>A, VAF 44%  ***SRSF2*** c.284C>A, VAF 44% |
